# Assessment of Knowledge, Attitude, and Practice of Sunlight Exposure of Infants among Mothers Attending in Governmental Health Facilities in Farta District, South Gondar Zone, North West Ethiopia, 2018

**DOI:** 10.1155/2019/2638190

**Published:** 2019-09-23

**Authors:** Haileyesus Gedamu, Yilkal Tafere

**Affiliations:** ^1^Nursing Department, Bahir Dar University, Bahir Dar, Ethiopia; ^2^Public Health Department, Debretabor University, Debretabor, Ethiopia

## Abstract

**Background:**

Sunlight exposure serves an important purpose in human bodies. It promotes good health and plays a major role in the production of the very essential vitamin, vitamin D. Vitamin D is important for the growth of healthy, normal bones. Research conducted in different areas suggested that daily exposure to sunshine remains the cheapest, safest, and most effective method of preventing rickets.

**Objective:**

To assess knowledge, attitude, and practice of infants to sunlight exposure among lactating women in Farta district, in 2018.

**Method:**

Institution based cross sectional study design was conducted among mothers attending the selected health center. Systematic sampling method was used to select individual respondents. Data were entered and analyzed by using SPSS version 20.

**Result:**

Among 357 respondents identified for the study, 95% (*n* = 339) was responding to the interview. Of the total respondents, 49.9%, 46%, and 45.7% of them had poor knowledge, unfavorable attitude, and poor practice about sunlight exposure, respectively.

**Conclusion and recommendation:**

The results of this study showed that almost half of the mothers had inadequate knowledge, attitude, and inadequate practice about sunlight exposure. Therefore, mothers need to be educated about the importance of sunlight exposure.

## 1. Introduction

Regular sunlight exposure increases the serotonin levels in the body, making it more active and alert. The most important advantage of exposure to sunlight is the ability to boost your body's vitamin D supply [1]. Exposing a child to sunlight through window panes for 10 min twice a day protects mild neonatal jaundice [2]. When people are exposed to sunlight in the morning, their nocturnal melatonin production occurs sooner, and they enter into sleep more easily at night. Melatonin production also shows a seasonal variation relative to the availability of light, with the hormone produced for a longer period in the winter than in the summer. Even if exposure of children to sunlight is important for the development, exposure to high sunlight is a risk factor in the development of skin cancers [3, 4].

Studies suggest that achieving and maintaining an adequate vitamin D blood serum level, as determined by a 25-hydroxyvitamin D (25(OH)D) blood test, is essential to the prevention and treatment of autoimmune diseases such as multiple sclerosis and type 1 diabetes. Additionally, vitamin D appears to play a protective role in cardiovascular health, various types of cancer, Autism, depression, schizophrenia, and respiratory conditions such as cystic fibrosis [5].

Recent knowledge suggests that the risk of some chronic diseases could be reduced if vitamin D intake and sun exposure of the population were increased. In some developed countries, the prevalence of rickets in the general population diminished after the emergence of dietary supplementation. However, in such countries, vitamin D deficiency rickets has re-emerged in recent years, particularly among groups with limited exposure to UVB-containing sunshine [6].

Previous studies indicate that insufficient exposure of sunshine was an important cause of rickets in children [7–10]. Studies indicate that regular exposure to sunshine is the most effective way of preventing the disease [8, 11, 13]. Health education has a vital role on behavioral change regarding infant's exposure to sunshine. Hence in the early 1960s, it was adopted as the main strategy to prevent rickets [13]. Evidence of world health organization indicate that worldwide one billion people have vitamin D deficiency [14]. A risk of rickets is dominant in children who live in crowded houses, which have no sun light [15].

Evidence shows that many people have low vitamin D levels and also there is a well-documented relationship between low vitamin D levels and poor bone health [16]. Beside these, studies in Gaza strip in 2010 among 340 cases and controls show only 65% have sunlight exposure. The prevalence of rickets and its determinant factor were educational status, occupation, number of deliveries, health education, and exclusive breastfeeding (EBF) [17].

Nutritional rickets are gaining the attention of public health professionals and individual clinicians worldwide as the disease remains an endemic problem in many developing countries and has re-emerged in a number of developed countries [18].

In middle east vitamin D deficiency and rickets continue to be a public health problem despite all year sunshine. In some parts of Asia, such as the northern parts of China (including Tibet), Mongolia, and Afghanistan rickets appears to be mainly due to vitamin D deficiency associated with the high latitude, cold winters, and limited skin exposure [19].

Vitamin D deficiency and nutritional rickets are still health problems in developing countries. Despite Turkey being in a geographical location with abundant sun light exposure, vitamin D deficiency continues to be a major health problem. Yearly incidence rates of vitamin D deficiency revealed in Turkey vary from 1.67% to 19%. Thus, sunning for bone development is still being advised [20]. In spite of adequate sunshine, vitamin D deficiency (nutritional) rickets has remained a common problem in the tropics and sub-tropics, including some of the most populated countries such as China, India, Nigeria, Egypt, Iran, and Turkey [21].

In Ethiopia, its prevalence was in the order of 30% in the 1950s and does not seem to have changed over the years. The main cause of nutritional rickets in Ethiopian children is lack of exposure to sunshine and not adequate intake of vitamin D. Lack of awareness and traditional beliefs are major causes for not exposing infants to sunshine [22].

A retrospective study in jimma university specialized hospital revealed that the prevalence of rickets was 10.5% [23]. In a research conducted in Addis Ababa town, four in ten children who had visited health institutions had rickets and the problem was higher in infants [24]. Other study conducted in jimma hospital showed that nearly one in ten children have rickets [25].

## 2. Methods

### 2.1. Study Design

Institutional based cross-sectional study design was conducted.

### 2.2. Study Area and Period

The survey was done from February 15, up to March 25, 2018, in Farta district, Amhara regional state. Farta district is located in the northwest part of Ethiopia and about 667 km from Addis Ababa and 99 km from Bahir Dar. The area is found at the altitude, which ranges from 2000 to 2500 meters above sea level and it consists of four major agro-ecological zones: 25% low land, 45% medium highland, 24% highland, and 6% gorge. The annual temperature ranges between 9 and 25 degree Celsius and the rainfall varies from 1250 mm in the lowlands to 1500 mm in the highland areas during summer.

### 2.3. Source Population

All lactating women whose child's age was less than 1 year and attended all health institutions of the Farta district for health care and immunization.

### 2.4. Study Population

All lactating women whose child's age was less than 1 year and attended selected health institutions of the Farta district for health care and immunization during the study period.

### 2.5. Inclusion and Exclusion Criteria

Inclusion criteria—All women who were lactating with a child less than 1 year.

Exclusion criteria—Mothers who were unable to communicate were excluded.

### 2.6. Sample Size Determination and Sample Selection

To determine the sample sizes required existence of estimated prevalence rates is compulsory's the prevalence of knowledge, attitude, and practice of sunlight exposure was 38.8% of Jimma university specialized hospital with an absolute precisions of ±5% and a statistical confidence of 95% [24].

The sample size is computed using the following formula.

(1) $$\begin{array}{c} n=\frac{Z^{2}p\left(1-p\right)}{d^{2}}\text{,} \end{array}$$

where, *n* = sample size; *z* = statistical certainty chosen; *p* = estimated prevalence level to be investigated; *q* = 1 − *p*; *d* = precision desired

(2) $$\begin{array}{c} n=\frac{\left(1.96\times 1.96\right)\left(.388\times .612\right)}{0.05\times 0.05}=365. \end{array}$$

The total number of infants in Farta district is 4973. Since this figure is below 10,000.

Use the following adjustment formula for the sample size:

(3) $$\begin{array}{c} n=\frac{n}{\left(1+n/N\right)}\text{,} \end{array}$$

where,

 
*n* = sample size for population of size above 10,000
 
*N* = number of infants in Farta district. Therefore,

(4) $$\begin{array}{c} n=\frac{365}{\left(1+365/4973\right)}\text{.} \end{array}$$

(5) $$\begin{array}{c} n=340. \end{array}$$

Taking 5% [17] non response rate the final sample size was **357**.

### 2.7. Sampling Technique and Procedure

The sampling method employed in this study was a systematic sampling method. That means proportional numbers of mothers were included into the sample from each selected health facility to make up a total sample size. Each study participant was selected using the systematic sampling technique in which every second client was interviewed in each health facility.

Proportional allocation of the study subjects of the four health facilities were as follows:

 
*n* in health facility = *N* in a health facility ∗nt
 
*N* total

where,

 
*n* in health facility = proportion of mothers with infants in a given health facility
  nt = Total sample size
 
*N* in a health facility = Number of mothers with infants in a given health facility
 
*N* total = Total number of mothers with infants in all health facilities.

### 2.8. Variable of the Study

#### 2.8.1. Dependent Variables

Knowledge, attitude, and practice of sunlight exposure of infant.

#### 2.8.2. Independent Variable

Age, occupation, income, educational status, no. of sibling and culture, place of delivery, antenatal care, and postnatal care.

### 2.9. Data Collection Procedures

A semi structured pre-tested questionnaire was prepared and translated into Amharic by an expert to ensure its consistency. A pretest study was also carried out in other health center to gain some useful feedbacks.

### 2.10. Data Quality Control

To ensure quality data collection, one day training was provided to data collector to make sure good interviewing techniques. The training was focused on how to complete the questionnaire, proper interview techniques, and proper asking. Through the data collection process, the researcher was closely supervised and checked for consistency and reliability of the data collected by interviewers.

### 2.11. Data Processing and Analysis

Data entry formats were designed by defining the variables and labeling values for categorical variables. Specifically coding of the questions and data entry format was prepared and made ready before the data was collected. The data were entered and analyzed using computerized SPSS version 20 software. The raw data were edited and checked to control entry. Table and figures were used to present the finding.

### 2.12. Ethical Clearance

Ethical clearance was first obtained from the research and publication office of Bahir Dar University. Ethical permission was also secured from, regional health bureau, South Gondar zonal health department & Farta district health office. Participation in the study was voluntary, and data were collected after obtaining permission from each participant.

## 3. Results

### 3.1. Socio Demographic Characteristics

Among the 357 mothers identified in the study, 339 (95%) responded to the interview, nearly all 323 (95.3%) were married. More than half, 176 (52%) of the women were in the age group 20–29. Almost all, 332 (98%) of respondents were orthodox and majority, 132 (38.8%) of child's age were under 4 months. More than two-thirds, 241 (71.1%) of maternal educational status were illiterate. More than half, 312 (91.9%) of their occupations were farmer. Almost all (70%) of respondents incomes were above five hundred Birr (Table 1).

### 3.2. Obstetrical History of Mothers' about Sunlight Exposure of Their Infant

Most, 306 (90.3%) of respondents had ANC follow up and the remaining 33 (9.7%) had no follow up. Among respondents who had ANC follow up, 113 (37%) of having four and above visit, only low proportion, 26 (8.6%) of women had one time visit. More than half, 180 (53%) of the mothers delivered at the health center. One hundred eighty eight (55.4%) of respondents had PNC follow up .Among respondents who had PNC follow up 79 (42%) of 188 respondents have a 1^st^ PNC visit and 60 (32%) had three visits (Table 2).

### 3.3. Level of Mothers' Knowledge about Sunlight Exposure of Their Infants

Nearly half (49.9%) of the respondents had knowledge of sunlight exposure to their infant. Of 183 respondents, 84 (46%) had knowledge of sunlight exposure every day and 39 (11.4%) two times per week. Nearly three quarters, 134 (73.2%) of 183 respondents had knowledge of sunlight exposure to infants before four hours and the remaining, 7 (2.2%) after four hours. Similarly, more than three quarters, 68 (29.8%) of 183 respondents had knowledge of sunlight exposure of infant in less than 30 min and the remaining, 29 (11.9%) for more than 30 min. More than half, 183 (53.98%) of respondents had information about sunlight exposure of infant and most of the respondents, 120 (54.6%) had got the information from a health professional (Table 3).

### 3.4. Practice of Respondents on Sunlight Exposure of Infant

Less than half of the participants (45.7%) were exposed their infants to sunlight. Only, 37 (15.7%) of 176 respondents started to expose their child to sunlight before 10 days and the remaining, 198 (84.3%) were after 10 days. Out of 176 respondents, about 80 (45.7%) participants exposed their child to sunlight every day and the remaining 96 (54.3%) were 5 up to 6 times a week and less. Most of, 169 (75%) of respondents covered their infant's body when they exposed (Table 4).

### 3.5. Attitude of Respondent on Sunlight Exposure of Infant

Of the total respondents, 46% agreed with an advantage of sunlight exposure of their infants. More than half, 176 (51.98%) of respondents were happy when they exposed/if they exposed their child to sunlight and the remaining, 163 (48%) felt anxiety and other. About 156 (46%) of respondents perceived consequences after they exposed or if they exposed their child to sunlight were to become strong and the remaining, 183 (54%) were to be healthy, helps to sleep, and others (Table 5).

## 4. Discussion

The aim of our study was to assess knowledge, practice, and attitude of sunlight exposure of lactating mothers to their infants in Farta district. Currently the government of Ethiopian gives emphasis to decreasing child morbidity and mortality. Therefore, assessing the knowledge, practice, and attitude of lactating mothers regarding exposing their infants to sunlight is one important aspect in maintaining children's health.

The proportion of respondents who reported that they had the information (knowledge) about sunlight exposure was 53.98%; the same studies done in Turkey and jimma town showed that 86.4% and 100% of mothers had information about sunlight exposure, respectively [20, 12]. Our research was lower than the study done in Turkey and jimma town; the possible reason for this might be different educational status of study participants about sunlight exposure.

Out of the total respondents who responded to the question “is sunlight exposure beneficial?”, 75.98% mentioned sunlight exposure was beneficial; our finding was lower than the study done in jimma town which was 99.68% [22]. The reason may be due to mothers in jimma town as mentioned above had 100% information about sunlight exposure and our finding was greater than the study done in sakarya which was 64.1% [21]. The possible reason for this may be mothers in sakarya were educated about harmful effects of sunlight exposure rather than benefits of sunlight exposure because they live in a high temperature region and they had fear of skin cancer. Seventy five percent of the respondents mentioned the most benefit of sunlight exposure was strengthening bone; our finding was similar to the same study done in jimma town which was 64.62% [12]. Regarding the best time to sunlight exposure of infants, 95.7% of the mothers mentioned it was in the morning; this is similar to the same study done in jimma town which was 100% [12].

Regarding the practice of sunlight exposure, 51.98% of mothers exposed their infants to sunlight. This finding is lower than the same study done in sakarya, which was 87.5% of mothers exposed their infants to sunlight [21]. The reason for this difference may be due to the adequate information mothers had in sakarya about sunlight exposure compared to mothers in Farta district. It was also greater than the study done in debre markos town which was 44.6% [28]. The possible explanation for this may be they were found in tropical regions which was increased prevalence of skin cancer; so due to fear of skin cancer, mothers may not have exposed their infants to sunlight.

This study showed that 15.7% of mothers started sunlight exposure of their babies between 0–15 days. This was lower than the study done in jimma town, 42.04% and debre markos town, 23.4% [22, 28]. This study also showed that 51.98% of the mothers exposed their infants to sunlight, but only 40% of mothers exposed their infants to sunlight daily and the rest of the mothers exposed their infants sometimes. This result was lower than the study done in jimma town which was 92.16% of mothers exposed daily [12]. The reason for this difference may be the community had more information and maybe there was a health education program for mothers in jimma.

In our study, 73.2% of mothers exposed their infants to sunlight in the range of time from 8 to 10 AM in the morning and 21.9% of participants exposed their infants with time duration of 15–20 min. It was lower than the study done in debre markos [28].

About 36.5% of respondents had feared to expose their infants to sunlight. Among these 15.2% of participants not exposed their infants to sunlight due to fear of the evil eye. This finding was higher than the study done in debre markos town which was 11.9% [28]. The possible explanation for this difference may be due to cultural differences between the two populations and differences in a study setting in Farta district; the majority of respondents were residing in rural areas, whereas in the study done in debre markose participants were residing in urban areas, which may have contributed to the observed difference.

For the current study, feeling of lactating women while they exposed their children, 53.98% were happy, 0.3% angry, 18.4% feel anxiety and 4.8% were other which was higher than with ethio-swedish children's hospitals. Respondents perceived consequences while they exposed their child to sunlight, 17.7% were makes healthy, 75% were made strong, 0.9 were helped to sleep, 27.7% were exposé the infant to cold (pneumonia), and 1.8 other which was higher than the result of ethio-swedish children's hospital [7].

However, this study does have some inherent limitations. Though there are wide ranges of factors which affect rickets among mothers attending governmental health institutions, only knowledge, attitude, and practice about sunlight exposure were addressed in this study. Hence, taking into consideration factors from the different contributors like calcium deficiency would have been important.

## 5. Conclusion

According to our study, participants had not good knowledge, practice, and attitude regarding sunlight exposure of infants. Therefore, health education focusing on the importance of sunlight exposure is important to improve knowledge, practice, and attitude of mother's sunlight exposure of their infants.

## Figures and Tables

**Table 1:** Socio demographic characteristics of respondents whose child age was less than 12 months in Farta district, Northwest Ethiopia, 2018.

Item	Category	Number	%
Age of mother mean = 28.8, SD = 6.2	<19 years	11	3.1
	20–29 years	176	52
	30–39 years	91	26.8
	40–49 years	61	18

Age of child in months mean = 5.99, SD = 3.25	<4	132	38.8
	5–8	113	33.4
	9–12	94	27.8

Gender of child	M	184	54.3
	F	155	44.7

Number of children	1	72	21.2
	2–5	218	64.3
	6–10	49	14.5

Religion of the mother	Orthodox	332	98
	Muslim	5	1.6
	Protestant	2	.4

Marital status	Single	0	.6
	Married	323	95.2
	Windowed	4	1.2
	Divorced	12	3.5

Educational status of the mother	Unable to read & write	241	71.1
	Able to read & write	54	15.9
	Primary school(1–8)	32	9.4
	Secondary school and above	12	3.5

Husband educational status	Unable to read & write	195	60.5
	Able to read & write	54	16.7
	Primary school(1–8)	49	15.2
	Secondary school and above	25	7.7

Monthly income	<500	102	30
	500–1000	143	42.3
	>1000	94	27.7

Maternal occupation	Farmer	288	85
	Civil servant	4	1.2
	Merchant	5	1.5
	Daily laborer	6	1.8
	House wife	36	10.6

Husband occupation	Farmer	275	85.2
	Civil servant	12	3.7
	Merchant	15	4.6
	Daily laborer	9	2.8
	Other	12	3.7

Mass media	Yes	190	55.9
	No	149	44.1

Type of mass media	Radio	161	84.9
	Television	26	15.1

**Table 2:** Obstetric history of women whose child age was less than 12 months in Farta woreda, Northwest Ethiopia, 2018.

Item	Category	Number	%
ANC visit	Yes	306	90.3
	No	33	9.7

Number of visit	One	26	8.6
	Two	97	31.7
	Three	70	22.9
	Four and above	113	37

Place of delivery	Home	54	15.9
	Health post	40	11.8
	Health center	180	53
	Hospital	65	19.2

PNC visit	Yes	188	55.4
	No	151	44.6

Number of visit	One	79	42
	Two	29	15.4
	Three	60	32
	Four	20	10.6

**Table 3:** Knowledge of respondents on sunlight exposure of infant in Farta district, Northwest Ethiopia, 2018.

Item	Category	Number	%
Mentioned as they knew the importance of sunlight exposure	Yes	183	53.98
	No	156	46.02

Number of day of sunlight exposure per week	Every day	84	46
	2	39	11.4
	3–4	48	26
	5–6	12	5

Starting day of sunlight exposure	Within 5 days	17	9.3
	5–10 days	20	8.3
	10–15 days	91	37.9
	After 15 days	55	46.7

Time of sunlight exposure	Before 16:00 h	134	73.2
	16:00–19:00 h	38	3.3
	19:00–22:00 h	11	.8

Duration of sunlight exposure	<15 min	49	26.7
	15–20	40	21.9
	20 min–1 h	54	29.5
	Above 1 h	35	19.1

Information on sunlight exposure	Yes	183	53.98
	No	156	46

Source of information on sunlight exposure	Mass media	12	5
	Health professional	100	54.6
	Neighbor	68	36.6
	Parents	3	1.1

**Table 4:** Practice of respondents on sunlight exposure of infant in Farta woreda, Northwest Ethiopia, 2018.

Item	Category	Number	%
Do you expose your child for sunlight	Yes	176	51.98
	No	163	48.02

Number of day of sunlight exposure per week	Every day	80	45.7
	2	28	15.9
	3–4	43	24.4
	5–6	25	10.6

Starting day of sunlight exposure	Within 5 days	17	7.2
	5–10 days	20	8.5
	10–15 days	91	38.7
	After 15 days	107	45.5

Time of sunlight exposure	Before 4 h	129	73
	4–7 h	38	21.5
	7–10 h	9	5.5

Duration of sunlight exposure	<15 min	59	33.5
	15–20	38	21.5
	20 min–1 h	69	38.5
	Above 1 h	10	5.7

Do you cover the infants body when you exposed	Yes	169	75
	No	56	25

Reason for you cover	To prevent skin damage	44	19.3
	To prevent from evil eye	98	55.7
	Other	27	25

Reason of respondents for not exposing	Fear of blackness	4	2.4
	Lack of knowledge	76	46.6
	Fear of evil eye	59	36.2
	Fear of pneumonia	5	3.1
	Lack of time	5	3.1

**Table 5:** Attitude of respondents on sunlight exposure of infant in Farta Woreda, Northwest Ethiopia, 2018.

Item	Category	Number	%
Sunlight exposure is Advantageous	Agree	139	41
	Disagree	155	45.7
	Strongly agree	17	10.98
	Strongly disagree	28	2.3

Feeling of respondents while they expose their child	Happy	176	51.98
	Anxiety	80	23.6
	Angry	3	.9
	Other	80	23.6

Perceived consequence of respondents on sunlight exposure of infant	To become healthy	60	17.7
	To become strong	156	46
	Helps to sleep	3	.9
	Exposes my child to cold/pneumonia	94	27.7
	Others	26	7.7

Thought that sunlight exposure cause skin cancer	Yes	203	60
	No	136	40

Thought that sunlight exposure cause nappy rash	Yes	237	70
	No	102	30

## Data Availability

The datasets generated during the current study are available from the corresponding author on reasonable request.
